# Wearables in rugby union: A protocol for multimodal digital sports-related concussion assessment

**DOI:** 10.1371/journal.pone.0261616

**Published:** 2021-12-22

**Authors:** Dylan Powell, Sam Stuart, Alan Godfrey

**Affiliations:** 1 Department of Computer and Information Sciences, Northumbria University, Newcastle-upon-Tyne, United Kingdom; 2 Department of Sport, Exercise and Rehabilitation, Northumbria University, Newcastle-upon-Tyne, United Kingdom; University of Rochester, UNITED STATES

## Abstract

**Background:**

Pragmatic challenges remain in the monitoring and return to play (RTP) decisions following suspected Sports Related Concussion (SRC). Reliance on traditional approaches (pen and paper) means players readiness for RTP is often based on self-reported symptom recognition as a marker for full physiological recovery. Non-digital approaches also limit opportunity for robust data analysis which may hinder understanding of the interconnected nature and relationships in deficit recovery. Digital approaches may provide more objectivity to measure and monitor impairments in SRC. Crucially, there is dearth of protocols for SRC assessment and digital devices have yet to be tested concurrently (multimodal) in SRC rugby union assessment. Here we propose a multimodal protocol for digital assessment in SRC, which could be used to enhance traditional sports concussion assessment approaches.

**Methods:**

We aim to use a repeated measures observational study utilising a battery of multimodal assessment tools (symptom, cognitive, visual, motor). We aim to recruit 200 rugby players (male n≈100 and female n≈100) from University Rugby Union teams and local amateur rugby clubs in the North East of England. The multimodal battery assessment used in this study will compare metrics between digital methods and against traditional assessment.

**Conclusion:**

This paper outlines a protocol for a multimodal approach for the use of digital technologies to augment traditional approaches to SRC, which may better inform RTP in rugby union. Findings may shed light on new ways of working with digital tools in SRC. Multimodal approaches may enhance understanding of the interconnected nature of impairments and provide insightful, more objective assessment and RTP in SRC.

**Clinical trial registration:**

NCT04938570. https://clinicaltrials.gov/ct2/results?cond=NCT04938570&term=&cntry=&state=&city=&dist=

## Introduction

Rugby union has the highest occurrence of Sports-Related Concussion (SRC, otherwise known as mild traumatic brain injury) of any contact sport [1]. The incidence of SRC (per 1000 hours) is 20.4 in professional rugby union, 16.6 in university rugby union and 4.7 national league rugby [2]. Consequently, participation in professional or amateur rugby union poses a considerable risk of sustaining injury [3–5]. SRC can cause a variety of motor and cognitive effects which typically resolve without any active treatment. However recognising SRC in a timely manner is essential to mitigate secondary injury and more severe adverse neurological impact [6–8]. Recent research highlighted the potential longer-term impact of inappropriate SRC management and links with the neurogenerative disease chronic traumatic encephalopathy [9,10]. Public health concerns that poorly managed SRC can cause harm to brain health and function in active and retired players, has driven demand for evidence-based research pertaining to diagnosis, monitoring and treatment [11].

### Sports related concussion monitoring and return to play

Despite the development of new objective physiological assessments in SRC such as biomarker screening [12], monitoring of SRC remains hugely challenging. Even in resource-rich environments where sufficient numbers of medical staff can better spot insidious mechanisms of injury associated with SRC, some diagnoses can be delayed or missed entirely [5,13,14]. In amateur environments where there may be one coach or first aider only, the detailed monitoring of SRC performed in professional contexts is not fully achievable. Therefore, in amateur environments with reduced medical provision, the binary approach of ’Recognise & Remove’ is adopted [15]. This involves permanently removing players suspected of SRC (e.g., clear contact to head) or if they display common signs and broad symptoms associated with SRC. The introduction of ‘Recognise and Remove’ approach has improved awareness and likely reduced the number of missed or SRC occurrences in low-resource, community environments.

SRC presents with a wide variety of signs and symptoms, which are often subtle, easily missed and may only become measurable in the hours and days following injury [16]. Typically this is followed by general management advice such as provision of head injury information leaflets only [14]. Therefore, significant challenges remain in the monitoring and objective or personalised return to play (RTP) procedures. There is inconsistency in what tests are performed during participants RTP, varying widely from neurocognitive testing to visual assessment [17,18]. Our previous work outlines the deficiencies in current traditional assessment methods [19,20]. The most commonly used mechanism administered by a health professional to inform RTP within amateur rugby union, is the 5^th^ version of the Sports Concussion Assessment Tool (SCAT5) questionnaire which tests symptoms, cognition, balance and vision [21,22]. The manual (pen and paper) and subjective nature of test like the SCAT5, mean formal SRC diagnosis, rehabilitation and RTP is based on clinical judgement [23], with a heavy weighting given to (self-reported) symptom assessment to determine readiness to play. Indeed, unions across the major rugby playing nations do not endorse the use of any novel technologies in RTP. Absence in regular data collection and lack of objective assessment means SRC recovery times and prognosis are highly variable among all players. This further highlights the need for valid and objective tools to aid diagnosis and monitoring [24].

### Progression to objective multimodal digital assessment

Non-invasive mobile wearable technologies have been used to objectively measure and monitor impairments in neurological injury [25,26]. Examples include visual assessment technologies to objectively monitor eye movements during laboratory tasks, assessing visual and cognitive processing [27,28]. In mobility assessment (e.g., balance, gait and turning), inertial wearables have successfully been used to track disease progression in Parkinson’s disease [29]. Wearables offer several advantages over traditional (non-mobile) methods of assessment. This includes the opportunity for passive monitoring, whereby continuous data can be collected on participants without their active attention or participation. Remote monitoring outside of clinic or laboratory can augment traditional assessment, and avoid ‘snapshot’ collection at episodic intervals [25,30]. Indeed, viewing SRC impairments in isolation could be futile and ignores the interconnected and related nature of SRC [22]. Wearables may provide continuous digital outcome measures, which can be easily compared and integrated with other impairments (e.g., cognitive function) [18]. SRC is considered a complex injury and will likely require a multimodal assessment approach to provide sufficient sensitivity for diagnosis and monitoring and enhanced understanding. Using a multimodal digital approach could provide objective outcome measures and robust data for more informed SRC assessment and monitoring [31,32]. However, to the authors knowledge, no multimodal protocols for digital assessment in SRC across rugby union have been published. As such there is a need to develop and refine multimodal protocol aims and methods, to translate technical research validation into clinical acceptance and application [33].

Here we propose a multimodal protocol and technical exploration for digital assessment and monitoring in SRC. We hypothesise that multimodal digital-based wearables will yield more objective data relevant to cognitive, gait, balance, turning, and visual metrics in those with s SRC compared to the traditional assessment method.

Primary aims:

1. Investigate use of multimodal digital-based wearables to capture objective data relevant to cognitive, gait, balance, turning, and visual metrics in those with SRC compared to a traditional assessment method.
2. Explore free-living mobility (balance, gait and turning) deficits with inertial wearable in those with SRC.

Secondary aims:

1. Explore the interaction and sex differences between cognitive, motor, visual and symptom characteristics, collected by wearables and questionnaires in those with SRC.
2. Consider practical and technical considerations of digital multimodal protocols in SRC.

## Materials and methods

### Study design

We aim to use a repeated measures observational study utilising a battery of SRC assessment tools (motor, visual and symptom assessment). The protocol was developed according to the Standard Protocol Items: Recommendations for Interventional Trials’ (SPIRIT) checklist [34], as appropriate. As this is a study protocol, no data has been included and conforms to PLOS data policy. The protocol was registered with clinicaltrials.gov (NCT04938570).

### Participants

University-level and amateur rugby players (males n≈100, and females n≈100) will be recruited and assessed over one season (June 2021 to August 2022). Participants will be stratified according to gender (males and females). The inclusion and exclusion criteria are outlined in Table 1. Those that have a mTBI/Concussion during the season must have a diagnosis of mTBI from a healthcare professional (physiotherapist or medic) based upon standard criteria or identified head injury from their contact sport governing body Although the number of SRC that will be observed during the season is not known, we will compare number of head injuries/SRC to our results from cohort baseline testing. Those that do not sustain a concussion will also have follow up testing at the end of the season, which will allow comparison between baseline and post-season.

**Table 1:** Inclusion and exclusion criteria.

Inclusion Criteria	Exclusion Criteria
≥18 years. Have minimal cognitive impairment, defined as a score between 0 and 8 on the Short-Blessed test for cognitive function. English as a first language or fluency. Those that have a mTBI/Concussion during the season must have a diagnosis of mTBI from a healthcare professional (physiotherapist or medic) based upon standard criteria or identified head injury from their contact sport governing body.	Medical history of a neurological illness that could grossly affect balance or coordination (such as. stroke, greater than mild TBI, lower-extremity amputation, recent lower extremity or spine orthopaedic injury requiring a profile). Be a pregnant female Have history of peripheral vestibular pathology or eye movement deficits. Be unable to abstain from medication/alcohol 24 hours in advance of testing

### Setting

Testing will be conducted at Clinical Gait Laboratory, Coach Lane Campus, Northumbria University, Newcastle upon Tyne and at the amateur rugby clubs in the North East of England.

### Recruitment

An ethics application was submitted to Northumbria University research ethics committee and approved June 2020 (23365). An amended ethics application (due to changes required from of the COVID-19 pandemic) was submitted to same ethics committee in October 2020 and approved in January 2021. Written, informed consent to participate will be obtained by all participants prior to each stage of the study in accordance with General Data Protection Regulations (GDPR). All Northumbria University Rugby Union Players will be invited to take part in the study. Additionally, local adult rugby union teams within 25 miles of Newcastle Upon Tyne will be invited to participate. An advertisement will be sent via email to local rugby clubs and the university rugby teams. Those interested will then be given a Participant Information Sheet (PIS) and a letter concerning the study with consent form. Inclusion and exclusion criteria are detailed in Table 1. In brief, all participants must be ≥18 years, have minimal cognitive impairment (Short-Blessed test 0 and 8), have fluency in English. Those excluded from participating in the study include anyone with a medical history that could grossly impact balance; stroke, severe TBI, amputation, vestibular pathology, alcohol addiction or substance abuse.

### Primary outcomes

The primary outcomes of this study are the proportion of players who have altered free-living, quality-based gait/walking patterns (e.g., gait speed), defined as micro gait characteristics measured by a digital inertial sensor-based wearable. Secondary outcomes are related to the change in free-living turning characteristics and clinical based visual data. Possible predictors for altered free-living micro gait patterns will include baseline assessment and acute SRC timeframe.

### Sample size calculation

The sample size calculation is based sample sizes from previous paper examining multimodal assessment, ~200 [18,35]. To determine the appropriate sample size (SS) for estimating the proportion of players we used the following formula (Eq 1) as previously described.


$$SS={\left(Z-score\right)}^{2}\times proportion\times \left(1–proportion\right)/{\left(margin\;of\;error\right)}^{2}$$ (1)

For a confidence level of 95%, α is 0.05 and the corresponding Z-value is 1.96. The sample proportion is unknown. We chose the number 0.50 (50%) because it takes the maximum spread into account. Consensus about the margin of error was achieved by joint discussion of the research group; a margin error of 0.075 (7.5%) was accepted. For a population size of 200 and a confidence level of 95%, α is 0.05 and the corresponding z-value is 1.96. Therefore, in total, 200 patients will be enrolled in the study to reach the necessary sample size.

### Participant stratification

**Fig 1:**
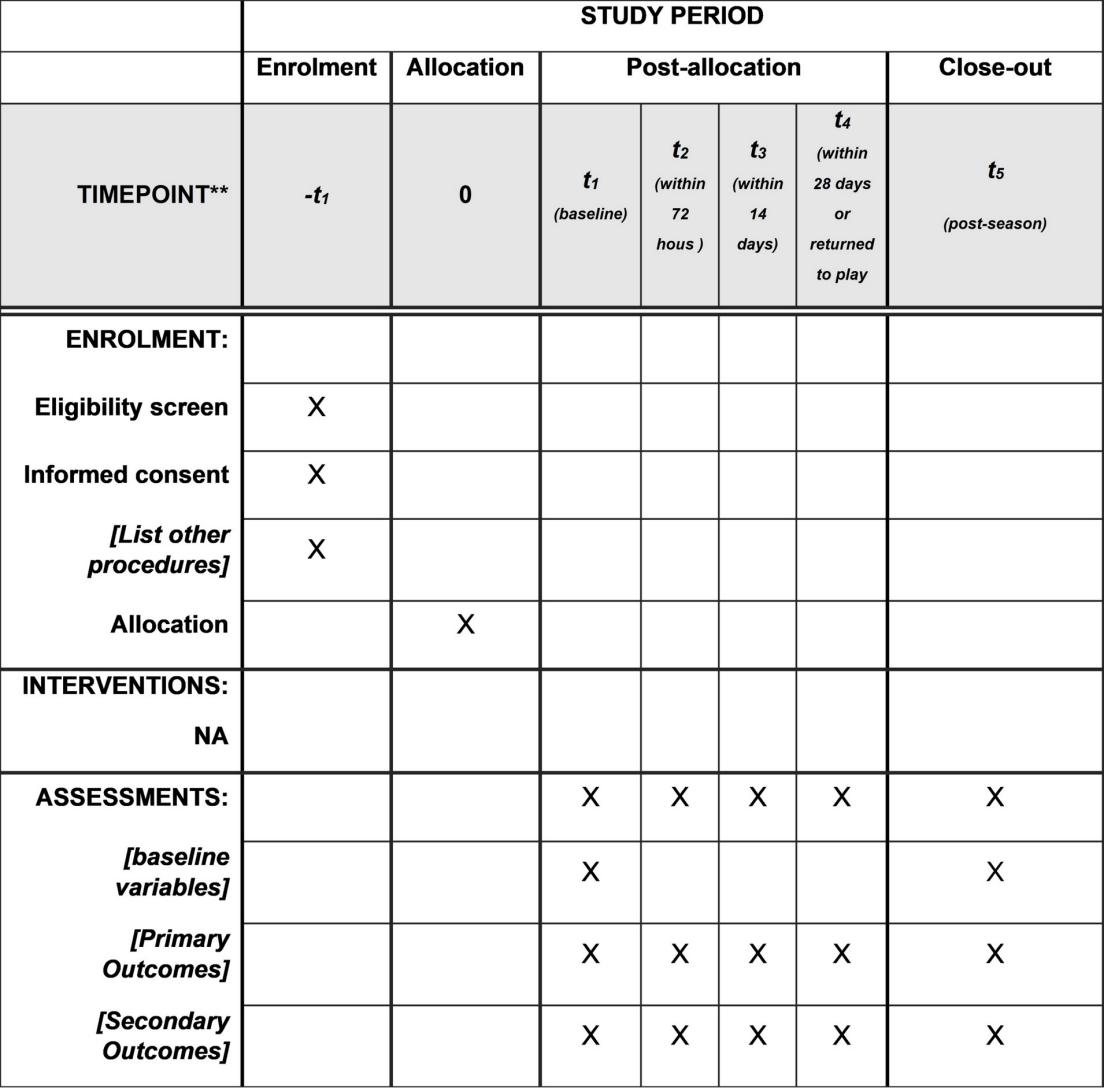
SPIRIT diagram, overall schedule and time commitment for trial participants.

All participants (male≈100, female≈100) who respond to the advertisement will complete baseline testing during pre-season and post-season. In-house university assessment will allow a clear pathway for concussed university players to be referred for post-SRC assessment. Local and amateur players who responded to the initial advertisement and sustain a SRC during the season can a) self-refer themselves (player) or b) be referred with consent by other personnel (physiotherapist, clinician, coaches,) into the study for testing. Testing availability for amateur players will be expanded (after 4:30pm Monday to Friday) to accommodate amateur player work/education commitments. Those diagnosed with SRC will be asked to attend a laboratory session with a subsequent free-living assessment at the following time frames post injury; within 72 hours post, 7–14 days post, once returned to play and post season. The overall schedule and time commitment for trial participants is depicted in Fig 1. (A more generic flow diagram depicting the schedule is presented in supporting material, S1 Fig).

### Anthropometric measures and screening

#### Test: Visual acuity (VA) eye chart and contrast sensitivity

VA is used to estimate degree of visual impairments in participants and will be measured binocularly using a standard eye chart. Participants will be asked to be seated or standing 4m from the chart. Participants will then be instructed to read aloud, starting from the top left and moving down the chart. The test is terminated if the participant makes two consecutive errors. Assessment will be done for right and then left eyes.

#### Test: Height, weight and leg length

Height (Bodysense Smart Scale, Eufy, USA) and weight (Seca 217, Seca Deutschland, Hamburg, Germany) will be measured for each participant. Participants leg length or sensor distance to ground will be measured [36], by a trained researcher/physiotherapist from posterior iliac spine to medial malleolus and used to inform inertial wearable algorithm analysis.

### Data collection: In the lab (traditional/reference assessments)

#### Test: Sports Concussion Assessment Tool 5th edition, SCAT5

*Estimated time*: *(10–15 minutes)*. The SCAT5 [37] is one of the most widely used assessment tools in aiding diagnosis and assessment measuring symptom scores [37], aspects of cognitive function (Standardised Assessment of Concussion [38] and balance function (modified balance error scoring system [39] via a pen and paper SCAT5 forms, Table 2.

**Table 2:** Sports Concussion Assessment Tool, 5^th^ version (SCAT5).

Assessment Domain	Component tests	Outcome Measures	Method of assessment
Symptom	Concussion Symptom Scale	Symptom severity (out of 22) Symptom total (out of 132)	Self-reported by player
Cognitive	Standardised Assessment of Concussion (SAC)	Orientation (out of 5) Immediate memory (out of 15 30) Concentration (out of 5) Delayed recall (out of 5 or 10)	Auditory/verbal assessment recorded on pen and paper
Neurological	Passive cervical movement	Pain (yes or no)	Subjectively assessed
	Finger nose test	Able to complete (yes or no)	Subjectively assessed
Visual	Horizontal Nystagmus	Double vision (yes or no)	Subjectively assessed
Balance	Tandem Gait	Able to complete (yes or no)	Subjectively assessed
	Modified Balance Error Scoring System (mBESS)	Total number of errors	Subjectively count number of errors

*Symptom*: The test measures aspects of symptom score and severity recorded across 22 symptoms self-reported by the player. A higher score indicates a more severe or worsened symptom profile (out of 132).

*Cognition*: The standardised assessment of concussion, is a mental status assessment previously developed [38] but now incorporated in the SCAT5 assessing individuals across immediate memory, concentration and delayed recall and recorded via the SCAT5 form.

*Balance*: The modified Balance Error Scoring System (mBESS) test [39] is an assessment protocol used to assess impairments in SRC [40]. The mBESS test assesses balance, postural stability across six different positions (double leg stance, single-leg stance, tandem stance) and tandem gait walking over 2.5–3 meters). Participants will be asked to maintain eyes closed, with hands placed on the iliac crest for each test’s duration (20 seconds). These tests are observed, and the number of errors counted. Errors are movements indicating a loss of balance or position such as; removing hands from iliac crest, stepping out with contralateral foot, stumbling or lifting forefoot or heel. The mBESS is assessed subjectively by the medical professional using a stopwatch and recorded using pen and paper. A higher error count indicating worse performance.

#### Test: Vestibular ocular motor screen (VOMS)

*Estimated Time*: *(5–10 minutes)*. The VOMS test includes a baseline measurement after which participants later verbally rate changes in headache, dizziness and nausea symptoms compared with their immediate baseline state on a scale from 0 (none) to 10 (severe) to determine if each of the tests provokes symptoms [41]. The test then measures impairments via this self-report across five sections (smooth pursuit, saccades, convergence, vestibular ocular reflex test and visual motion sensitivity test), Table 3. Testing will be conducted on a standard height of chair (45cm) at a distance of 90-100cm away from the stimuli.

**Table 3:** Laboratory testing: Multimodal approach for sports related concussion assessment.

Assessment Domain	Test	Digital Approach	Digital Technology	Primary Outcome Measures	Time Commitment
Cognitive	Reaction Time	Computerised neurocognitive testing	Brain Gauge. Cortical Metrics, USA^1^	Reaction Time & reaction time variability (milliseconds)	10–15 minutes
	Amplitude Discrimination			Simultaneous and sequential amplitude discrimination (microns)	
Balance	SCAT 5^5^ (Modified Balance Error Scoring System)	Wearable Inertial Measurement Units	MoveMonitor, McRoberts, UK^2^	Postural stability characteristics Sway (speed at which the centre-of-pressure moves) Root mean square (average variance signal captured) Jerk (the rate of change of acceleration from signal)	10–15 minutes
Gait & Turning	SCAT 5^5^ Lab: (Tandem Walk)	Wearable Inertial Measurement Units	MoveMonitor, McRoberts, UK^2^	**Gait characteristics** Mean stance time (seconds, s) Mean step time (s) Mean stride time (s) Mean swing time (s) Mean stride length (cm) Mean stride velocity (cms-1) **Turning characteristics** Number of turns per hour (n) Turn Angle(°) Turn Duration(seconds) Turn Velocity (°/seconds)	10–15 minutes
	Lab: Two Minute Walk Test				
	High Level Mobility Assessment Tool (HiMAT)				
Visual	SCAT 5^5^ (Horizontal Nystagmus test)	Mobile Eye Tracker	Pupil Labs, Core Eye Tracker, Germany^3^ Tobii Pro Glasses 2^4^ (100Hz, Tobii Technology Inc., VA, USA)	**Visual characteristics** Mean and variability of fixations, saccades and smooth pursuit	10–15 minutes
	Visual Oculomotor Screen				
Questionnaires	Neck Disability Index	Mobile application/secure questionnaire	PC or Tablet	Symptom Severity and symptom number	20–30 minutes
	Lower Extremity Function Scale			Symptom Severity and symptom number	
	International Physical Activity Questionnaire			Self-reported activity levels	
	Dizziness Handicap Inventory			Symptom Severity and symptom number	
	Neurosymptom Inventory Index			Symptom Severity and symptom number	

^1^ https://www.corticalmetrics.com/.

^2^ https://www.mcroberts.nl/products/movemonitor/.

^3^ https://pupil-labs.com/products/core/.

^4^ https://www.tobiipro.com/product-listing/tobii-pro-glasses-2/.

^5^ https://bjsm.bmj.com/content/bjsports/early/2017/04/26/bjsports-2017-097506SCAT5.full.pdf.

#### Test: Two-minute walk test

*Estimated Time*: *(5–10 minutes)*. Participants will be asked to complete two-minutes of continuous walking [42,43] at self-selected, normal walking speed over 8m with 180° turns, single and dual-task (Table 3). Cognitive measurement to determine dual task will be conducted prior to any walking. The dual-task will involve the backwards digit span [44], which will be set to the maximal amount of numbers recalled in sitting. The first walking trial will be single task walking. Secondly for dual task, the participant will hear a series of numbers while walking and repeat the numbers in backwards order while walking. Participants will be instructed to concentrate on both tasks equally.

#### Test: High Level Mobility Assessment Tool, HiMAT

*Estimated Time*: *(5–15 minutes)*. HiMAT is a standardised outcome measure used to quantify motor performance in individuals with high-level balance and mobility deficits [45]. The HiMAT is scored over 13 items derived from expert clinicians’ opinions and from existing multi-dimensional mobility scales. which includes tasks such as: backwards tandem walking, Walk over obstacle, Up/downstairs.

### Digital technologies

We will use traditional approaches but overlay those approaches with digital technologies to provide more objective outcome measures.

#### Digital neurocognitive tests

Conducted with the Brain Gauge Pro, Cortical Metrics, Chapel Hill, NC, USA (www.corticalmetrics.com). Testing takes approximately 8 minutes and is completed with participants sitting at a laptop [46,47]. Two computer mouse probes on the device provide a stimulus through vibration (25-50Hz) for participants index (D2) and third (D3) fingers. Participants are asked to respond by pressing their D2 and D3 according to specific tests. Outcomes calculated by the technology are reaction time (RT) measured in milliseconds, sequential, simultaneous amplitude discrimination (measured in microns) and reaction time variability.

#### Wearable eye-tracking

Conducted with the wearable eye tracker (Pupil Labs, Core Eye Tracker, Berlin, Germany. 160×51mm, high speed 120hz and 200hz https://pupil-labs.com/products/core/) and Tobii Pro Glasses 2 (100Hz, Tobii Technology Inc., VA, USA www.tobiipro.com/product-listing/tobii-pro-glasses-2/) which have shown to have good accuracy and showed the least error accuracy error overall in comparison with three other models of wearable eye-trackers [48]. The wearable eye-tracker in this protocol will be compared to a subjective test (VOMS), which has been clinically adopted in neurological assessment and will be used a reference standard [41,49,50].

#### Inertial wearable

The MoveMonitor (McRoberts, Netherlands; 106.6×58×11.5mm, 55g www.mcroberts.nl/products/movemonitor) comprises an accelerometer (+/- 100Hz) and gyroscope (+/- 8g) tri-axial sensors and is worn on the fifth lumbar vertebrae (L5), attached with an elastic strap. The wearable has been used extensively for functional and mobility monitoring in neurological disorders and is considered a valid technology which can capture data in controlled/lab/clinic and free-living environments [51–54]. This will be used to compare against traditional methods of balance, gait and turning assessment in the mBESS and walking tasks (lab and free-living assessment).

### Instrumentation: Using digital technologies during traditional assessments

#### VOMS and eye tracking

Due to the test’s subjective outcomes (provocation of non-specific symptoms), the VOMS cannot be used in isolation to diagnose SRC. Wearable eye trackers may provide an objective method of instrumenting traditional subjective tests like the VOMS and yield enhanced metrics on fixations, saccades and smooth pursuit [55,56]. We will use the Pupil Labs, Core eye tracker or Tobii Pro Glasses 2 while comparing the traditional VOMS test results across three main movements, fixations, saccades and smooth pursuits. Data is wirelessly transferred to Pupil Labs/Tobi proprietary software and stored locally. Data will then be stored on a secure Further analysis of these will be made using a custom-made MATLAB^®^ (MathWorks Inc, Massachusetts, USA) algorithm as previously described [49,57].

#### Balance, gait and turning

By instrumented digital approaches such as use of inertial sensor-based wearables, detection of subtle deficits may be detected. Indeed, the instrumentation of the balance error scoring system (BESS) has been shown to have superior diagnostic classification compared to traditional balance tests in concussion/mTBI [58]. Data will be download to PC or laptop via USB and uploaded to a secure database or file storage and analysed. Movement bouts will calculated for lab and free-living balance, gait and turning characteristics using bespoke MATLAB^®^ algorithms [59,60]. Free-living data will be initially processed using two separate custom-made and validated MATLAB^®^ algorithms to estimate free-living balance (e.g., jerk, sway), gait (e.g. mean step time, stance time variability) and turning (e.g., peak velocity, turn duration) characteristics [60–63].

Differences in gait between single and dual task will be examined rather than dual task cost. Absolute dual-task differences between groups (healthy vs concussed) will be examined to investigate if objectively measured dual-task walking could be a useful assessment for concussion, which will be compared to the use of single-task gait outcomes.

### Data collection: Beyond the lab

At present wearable laboratory-based motor assessment in SRC only offers a snapshot assessment. Little research has focussed on participants motor assessment outside of the laboratory at episodic intervals of assessment. To overcome this limitation, testing could be better utilised through constant remote evaluation in free-living environments. This would mitigate the need for the clinician to be present and would allow a higher frequency of testing within the players own environment [64]. Although testing in the latter would be conducted in less controlled conditions, there is considerable value in conducting testing in remote, real-world/free-living as s/he would be within habitual conditions [25,65,66].

#### Test: Free-living gait and turning assessment (7 days)

After laboratory testing participants will wear the MoveMonitor (L5) continuously for 7-days (weekdays and weekend to examine daily habitual fluctuations). Participants will be instructed how to take off and reattach the device for general hygiene purposes and return the device at their next laboratory visit. Free-living balance, gait and turning data will be segmented from raw (sample level) data and analysed to generate clinically relevant spatial and temporal outcomes to examine habitual motor and behavioural characteristics as previously described, Table 4 [59,60]. Application and evaluation of conceptual models previously described [66–68] will be applied to provide better insight to habitual player recovery, which may better inform RTP.

**Table 4:** Data collection: Beyond the lab.

Assessment Domain	Digital Approach	Digital Technology	Primary Outcome Measures	Time
Balance, gait & turning	Wearable Inertial Measurement Units	MoveMonitor, McRoberts, UK	**Balance** Root mean square (m/s^2^), Jerk (m^2^/s^5^), Sway (area, mm^2^/s^5^) **Gait** Mean stance time (seconds, s) Mean step time (s) Mean stride time (s) Mean swing time (s) Mean stride length (cm) **Turning** Number of turns per hour (n) Turn Angle (°) Turn Duration (seconds) Turn Velocity (°/seconds)	7 days
Symptom (SCAT5 Symptom)	Mobile application/secure questionnaire	PC or Tablet	Symptom severity (out of 22) Symptom total (out of 132)	5–10 minutes

SCAT5: Sports Concussion Assessment Tool 5.

#### Test: Concussion symptom checklist, SCAT5

*Technology*: *Mobile application/secure questionnaire*. Participants will complete symptom assessment daily throughout their RTP, via a secure mobile application or questionnaire. This will be from the concussion symptom scale as part of the SCAT5 [37].

### Digital outcomes (primary)

#### Cognitive characteristics

Reaction time tests how quickly participants can respond to stimuli. Reaction time variability is a measure of how quickly participants fatigue or concentrate [46,47]. Amplitude discrimination tests how well participants brain can differentiate between similar stimuli. These will be tested across all participants and tracked across different time points of recovery.

#### Balance (postural), gait and turning characteristics

The inertial balance, gait and turning characteristics will be estimations from the MoveMonitor. The balance (postural control tasks, BESS) include root mean square (m/s^2^), (root mean square of signal), Jerk (m^2^/s^5^), (first derivative of acceleration signal) and Sway (area, mm^2^/s^5^). Gait characteristics include step time (s), stride time (s), swing time (s), stance time (s), step length (m), step velocity (ms^-1^). Those comprehensive gait measures will be assess upon division into four original domains (pace, rhythm, variability and turning) based on the previously described model [68,69]. Turning characteristics include number of turns per hour (n), turn angle (°), turn duration (s) and turn velocity (°/s), Table 4.

#### Visual characteristics

As outlined in the visual oculomotor screening test, we will be comparing traditional VOMS versus the eye-trackers calculations for; (1) smooth pursuit, (2) horizontal and vertical saccades, (3) near point of convergence (NPC) distance, (4) horizontal vestibular ocular reflex (VOR), and (5) visual motion sensitivity (VMS) from the visual oculomotor screen.

### Secondary outcomes

#### Questionnaire #1: Neck disability index

*Estimated Time*: *(5minutes)*. The neck disability index (NDI) is a patient recorded functional status questionnaire [70] with 10 items (pain, personal care, lifting, reading, headaches, concentration, work, driving, sleeping and recreation). The NDI a commonly used self-reporting measure for neck pain which will be monitored across the study. This will be given to participants at each testing session and used to compare specific neck pain responses at baseline and at various stages in recovery from SRC.

#### Questionnaire #2: Lower extremity function scale

*Estimated Time*: *(5minutes)*. The lower extremity functional scale (LEFS) is a questionnaire containing 20 questions about a person’s ability to perform everyday tasks [71]. Clinicians can use the LEFS as a measure of patients’ initial function, ongoing progress and outcome, as well as to set functional goals. The LEFS can be used to evaluate the functional impairment of a patient with a disorder of one or both lower extremities and can be used to monitor the patient injuries progress over time. This will be used to account for injuries that may negatively impact gait and influence any changes measured.

#### Questionnaire #3 dizziness handicap inventory

The dizziness handicap inventory (DHI) is a 25 item self-report questionnaire designed to assess perceived dizziness affecting function [72]. The DHI will be used as secondary outcomes and compared between healthy and concussed individuals.

#### Questionnaire #4 neurosymptom inventory index

The Neurobehavioral Symptom Inventory (NSI) is a self-reported evaluation tool [73] frequently completed after a mild traumatic brain injury (mTBI). This will be used to monitor and measure post-concussion symptom changes over time and between healthy and non-concussed individuals.

#### Questionnaire #5 international physical activity questionnaire–short form

The development of an international measure for physical activity commenced in Geneva in 1998 and was followed by extensive reliability and validity testing undertaken across 12 countries [74]. This will be used as a reference standard to compare the participants self-reported activity levels.

### Statistical analysis

This is an exploratory study consisting of two groups. To the authors knowledge, there has yet to be a comprehensive free-living analysis of participants with SRC in rugby union. However, there have been analyses of non-sporting concussion/mTBI. Previous non-sporting studies have used datasets of 30–100 individuals [59]. Therefore, our anticipated dataset size of ~200 individuals, will provide greater statistical power to quantify between-group differences and detect small differences in visual, motor and symptom metrics. The multimodal battery of assessment used in this study will compare metrics between wearable systems and against traditional assessment methods. Data will be analysed in SPSS (v23, IBM) and R studio (R. RStudio, Boston, MA, USA). All data will be checked for normality distributed with Shapiro-Wilks tests before conducting parametric tests. Independent t-tests will be performed comparing demographic information between concussion and non-concussed groups. Anonymised data will be made available on reasonable request.

#### Primary analyses

The study aims will be explored with the analysis below.

1. *Investigate use of multimodal digital-based wearables to capture objective data relevant to cognitive*, *balance*, *gait*, *turning and visual metrics in those with SRC compared to a traditional assessment method*.
  Paired sample t-tests will be used to assess differences in group means for laboratory-based gait and balance assessment (mBESS, HiMAT, Two-minute walk -test, single and dual-task) and visual (VOMS) between healthy and SRC groups. To examine differences in SRC laboratory and free-living mobility across multiple recovery time points we will used multivariate analysis of covariance (MANOVA). To determine which features of each assessment domain (visual, motor, symptom) is best to distinguish SRC from healthy we will use receiver operating characteristic (ROC) and area under the curve (AUC).
2. *Investigate free-living mobility (gait characteristics) in those with sports*-*related concussion (SRC)*
  Between groups (concussed or non-concussed) differences in macro/micro gait and turning characteristics will be analysed with covariance (gender and age) for pre and post-season, free-living motor assessment and linear mixed models to further examine concussed player time-points and recovery.

### Secondary analyses

1. *Explore the interaction and sex differences between cognitive*, *motor*, *visual and symptom characteristics*, *collected by wearables & questionnaires in those with SRC*.
  We will use Pearson’s correlation analysis heatmap to explore the relationship between mobility, visual and self-reported symptoms in mTBI/SRC and across sex. Thus, this component of the interaction analysis will be data-driven, rather than hypothesis-driven. Statistical significance will be determined at p < 0.05 unless otherwise stated. Principal Component Analysis will be used to compare those with SRC history and no SRC concussion history across cognitive, visual and motor impairments. To assess and deduce if there are distinct groupings or clustering in the various cognitive, visual and motor characteristics.
2. *Consider practical and technical considerations of digital multimodal protocols in SRC*.
  Feedback will be collected from participants on usability of wearables during laboratory and free-living assessment using the system usability scale [75]. This will be analysed and compared across groups and time points in recovery.

## Discussion

Here we provide a protocol for multimodal objective SRC assessment, with a focus on wearable technologies. At present there is no gold standard or proposed method for SRC assessment, currently impairments are often viewed in isolation and not interconnected. This protocol will allow consideration of the combined and interactive impact of SRC on gait, cognition and vision and symptom recognition using wearables to collect objective data in university rugby union. This multimodal assessment paradigm distinguishes therefore itself from other work in the field. To the authors knowledge, no research has examined free-living gait in SRC among university rugby players. Furthermore, there hasn’t been attempts explore visual and motor impairments concurrently in laboratory and free-living environments. Therefore, the development and synthesis of this multimodal protocol would provide an important step in quantitively monitoring SRC motor and visual impairments and begin preliminary analyses of multimodal assessment in SRC.

This protocol does carry some limitations. Firstly, there are several equivalent technologies that could be deployed or tested across each component test (cognitive, motor and visual). However, given the lack of multimodal protocols in SRC, we feel the proposed manuscript provides a starting point to work on and develop in future research. Secondly, although we aim to have participants with SRC assessed within 72 hours of injury, this may not be feasible in all cases. Likewise follow up once returned to play, may not be always feasible if there are chronic issues associated with return to play and extended time lapse post injury. These limitations and solutions may become apparent when practically tested.

## Conclusion

Current SRC assessment focusses on impairments viewed in isolation, ignoring the interconnected nature and spectrum of SRC. As such, reliance on traditional methods of assessment and monitoring in SRC is limiting our understanding. Multimodal digital technologies can measure and monitor impairments non-invasively more informed assessments [25] in neurological injury [26]. By implementing a multimodal digital approach, a more objective and robust health profile could prevail. Additionally, with an increased frequency of testing, a greater insight into SRC progression and recovery may be possible. This combination of data (cognitive, gait and visual assessment) may uncover mechanistic interactions, showing trends between different impairments to infer new recovery patterns. Here the proposed multimodal protocol for digital assessment in SRC, could be used in conjunction and enhance the current sports concussion assessment tool approaches and may provide an important first step towards clinical deployment.

## Supporting information

S1 Fig Schematic/Flow-diagram of protocol. (DOCX)
