# Supplementary figures and images for: Wearables in rugby union: A protocol for multimodal digital sports-related concussion assessment
Source: PLoS One. 2021 Dec 22;16(12):e0261616. doi: 10.1371/journal.pone.0261616 (PMC8694415; doi:10.1371/journal.pone.0261616)

**Supporting material**


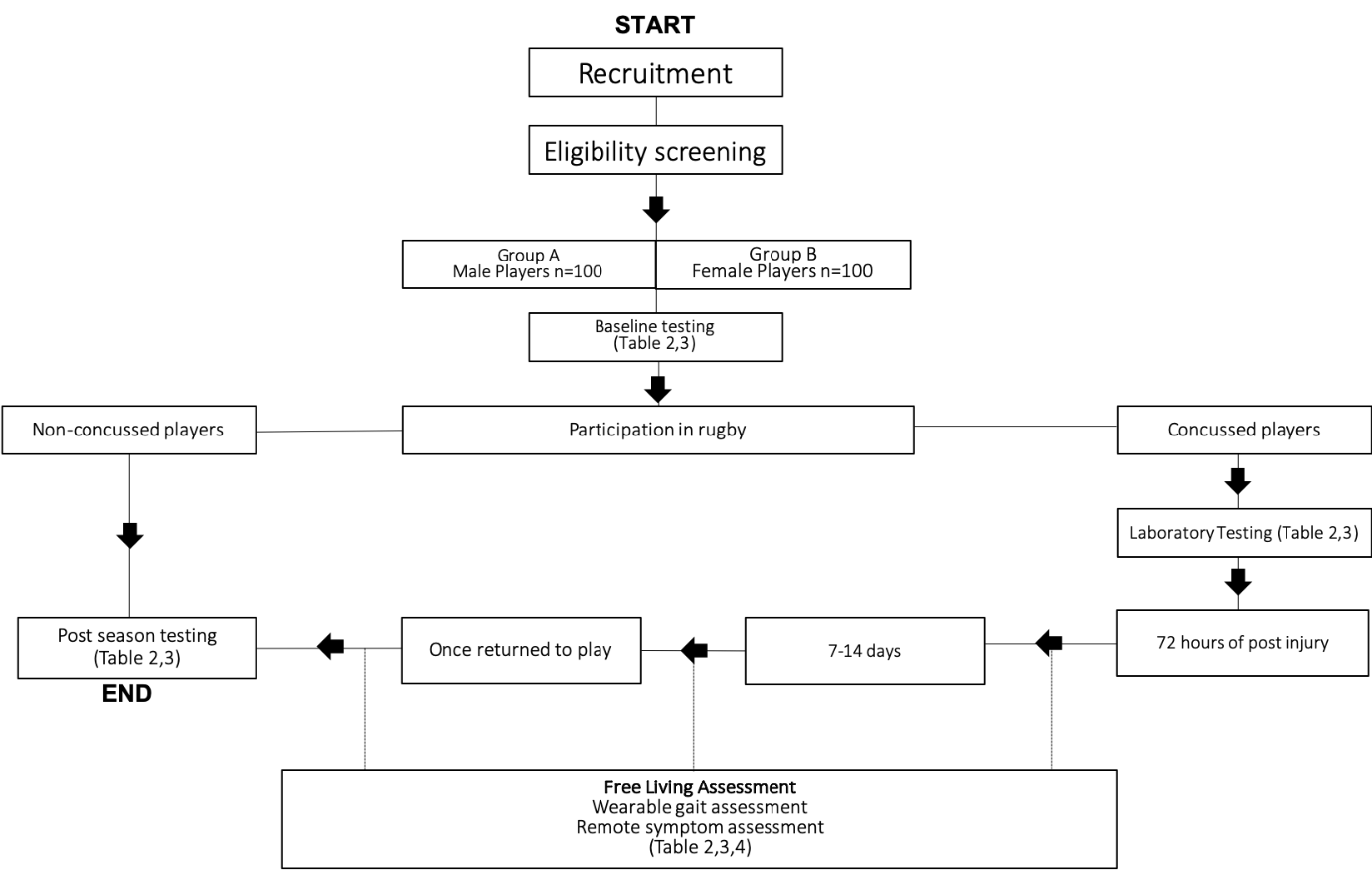


**S1 Fig:** Schematic/flow-diagram of protocol

Supplement: S1 Fig — (DOCX) [file pone.0261616.s001.docx]
